# Inference of Surface Membrane Factors of HIV-1 Infection through Functional Interaction Networks

**DOI:** 10.1371/journal.pone.0013139

**Published:** 2010-10-12

**Authors:** Samira Jaeger, Gokhan Ertaylan, David van Dijk, Ulf Leser, Peter Sloot

**Affiliations:** 1 Knowledge Management in Bioinformatics, Humboldt-Universität Berlin, Berlin, Germany; 2 Algorithmic Computational Biology, Centrum Wiskunde and Informatica, Amsterdam, The Netherlands; 3 Computational Science, University of Amsterdam, Amsterdam, The Netherlands; University of California Los Angeles, United States of America

## Abstract

**Background:**

HIV infection affects the populations of T helper cells, dendritic cells and macrophages. Moreover, it has a serious impact on the central nervous system. It is yet not clear whether this list is complete and why specifically those cell types are affected. To address this question, we have developed a method to identify cellular surface proteins that permit, mediate or enhance HIV infection in different cell/tissue types in HIV-infected individuals. Receptors associated with HIV infection share common functions and domains and are involved in similar cellular processes. These properties are exploited by bioinformatics techniques to predict novel cell surface proteins that potentially interact with HIV.

**Methodology/Principal Findings:**

We compiled a set of surface membrane proteins (SMP) that are known to interact with HIV. This set is extended by proteins that have direct interaction and share functional similarity. This resulted in a comprehensive network around the initial SMP set. Using network centrality analysis we predict novel surface membrane factors from the annotated network. We identify 21 surface membrane factors, among which three have confirmed functions in HIV infection, seven have been identified by at least two other studies, and eleven are novel predictions and thus excellent targets for experimental investigation.

**Conclusions:**

Determining to what extent HIV can interact with human SMPs is an important step towards understanding patient specific disease progression. Using various bioinformatics techniques, we generate a set of surface membrane factors that constitutes a well-founded starting point for experimental testing of cell/tissue susceptibility of different HIV strains as well as for cohort studies evaluating patient specific disease progression.

## Introduction

One of the important characteristics of Human Immunodeficiency Virus (HIV) is its ability to interact with many cell types and its capacity to alter the function of chemokines that otherwise work in harmony with the immune system. This interaction depends on the phenotype of the virus, the receptor type residing on the cell as well as the chemokines present in the environment. The main factor determining its complex interaction profile is HIV's highly interactive proteome. Structurally, its genome has evolved to interact with many human proteins from various cellular pathways. Therefore, each infectious virion consists of viral proteins, such as Tat, Gp120 or Nef, which interact with proteins inside and outside the cell [1]–[3].

Another contributor to this complex behavior is the high degree of phenotypic variation in the HIV population *in-vivo* [4]. Interestingly, each transmission event (between individuals) introduces an evolutionary bottleneck since the majority of new infections is usually initiated with a single virus [5].

Typically, HIV infection is thought to originate from the contact of genital epithelia with the infectious virions. It has been suggested that Langerhans cells and resident dendritic cells of stratified squamous epithelia serve as the initial targets of HIV infection [6], [7]. Virions are mobilized to the lymph nodes either via attachment of the HIV Gp120 to the DC-SIGN receptor expressed on dendritic cells (DCs) [6] or by direct infection of DCs within epithelia via CD4 and CCR5 receptors [7]. In the lymph nodes virions are transferred to CD4+ T cells and macrophages.

Moreover, soluble Gp120 binds to Immunoglobulin-E on innate immune system cells, such as basophils, mast cells and monocytes, and induces the secretion of cytokines thereby causing further activation of type-2 T-helper cells (Th2), the primary targets of HIV-1 infection [8]. The system-wide activation of CD4+ T cells results in an increased number of infected cells and high viral reproduction that leads to viral peaks observed in the primary stages of the infection. This translates into virus populations, which essentially are genotypically related cloud(s) of phenotypes (or quasispecies). The infection, which has been ignited with a relatively small number of virions, then spreads to other tissue types harbouring immune system cells, such as CD4+CCR5+CCR3+ microglia and macrophages [9], or hMR+ astrocytes [10], megakaryocytes [11] and monocytes [12].

A puzzling fact is that the cell types which are targets of HIV infection have different receptor expression profiles and do not necessarily harbor main co-receptors CCR5 or CXCR4. For instance, in a clinical study with a heterozygote CCR5-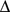
32 (CCR5 delta 32) individual (which gives partial resistance to infection via CCR5 tropic viruses) a wide range of co-receptor usage is observed, suggesting the involvement of other surface membrane factors [13].

Furthermore, binding of HIV to cell surface factors other than CD4 and chemokine receptors does not always permit viral entry but leads to endocytosis of the viral particles. This promotes relocation of the infectious virions, future trans-infection of adjacent cells [14] and leads to the activation of the immune system. Therefore, it is imperative to bear in mind that there are surface membrane factors interacting with HIV proteins, hence affecting the course of infection indirectly.

Another important point regarding surface membrane proteins is that their interactions with HIV-1 proteins are not only restricted to the extracellular environment. Events taking place inside and outside the cell membrane are neither decoupled processes, nor mutually exclusive. In vitro studies with HIV-1 protein Tat have shown that Tat is able to induce the intrinsic pathway of apoptosis in a number of human cell lines in addition to up-regulating the expression of co-receptor CCR5 and the interleukin-2 (IL-2) in HIV-1-infected cells. Extracellular Tat has also been shown to induce neuronal death by binding to the lipoprotein receptor-related protein (LRP) (see Romani *et al.* [15] for an extended review).

Although many steps of the virus life cycle have been unraveled and 24 distinct drugs targeted against HIV have been approved, all efforts to achieve an overall eradication of the virus have turned out to be ineffective [16]. However, life expectancy under highly active antiretroviral therapy (HAART) treatment has been extended to 21.5 years [17].

### The missing piece of the puzzle

These observations lead to the following questions: What is the extent of surface membrane factors contributing to HIV-1 infection and how do they influence the outcome of the treatment?

HIV exploits the existing signaling and regulatory pathways in its host. The different receptors or surface membrane proteins that are targeted in different cell types are likely to be involved in the same (or closely related) functional pathways, because the range of processes and pathways available to the virus is limited. The complexity in finding the right factors arises from the fact that there are several hundreds of surface membrane proteins expressed on a wide variety of cells.

Experimental testing of hundreds of targets from numerous pathways is not feasible. Therefore, we developed a computational approach that generates high quality hypotheses for wet-lab experiments with the aim to identify surface membrane host factors contributing to HIV-1 disease outcome. We adapt a strategy from disease gene discovery that is based on protein interaction, network centrality and functional similarity to receptors that are known to interact with HIV. We infer promising candidates using measures of centrality in the emerging network of proteins. This method reproduces reported factors, such as CCR1, CCBP2 and CD97, but also results in a list of promising proteins that likely affect the progression of the infection.

## Materials and Methods

We designed a method to identify uncharacterized surface membrane factors interacting with HIV. We employ a ranking strategy based on network centrality that uses documented HIV receptors, human protein interaction data and protein functions. The algorithm is partially adapted from disease gene identification strategies that infer gene-disease associations from similarity networks and their properties. Its underlying principle is based on the assumption that the most central genes/proteins in a specific disease network are likely to be related to the disease [18], [19].

### Conceptual design

**Figure 1:**
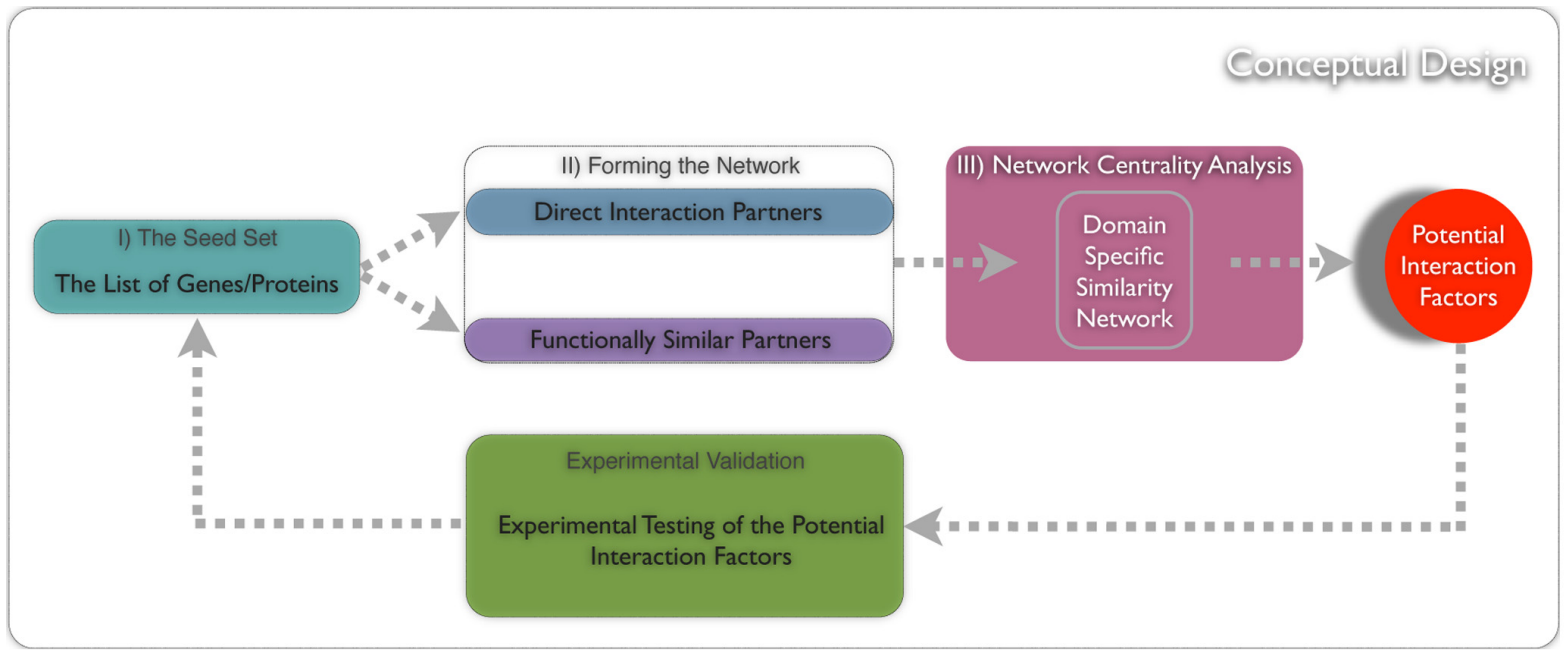
Conceptual design of our prediction framework. The method consists of three components. I) Compiling “the seed set” from genes/proteins sharing specific characteristic of interest; II) Forming a network by including direct interaction partners described in database(s) and/or functionally similar partners; III) Network centrality analysis on the domain-specific similarity network to obtain potential interaction factors (PIFs). The final step of such an analysis is the experimental validation of PIFs. Confirmed PIFs then can be included in the seed set and the steps I-III can be repeated for identifying new PIFs.

For identifying novel surface membrane factors we developed a generic framework that infers candidate genes or proteins based on their similarity to a set of reported genes or gene products of interest. The general workflow of this framework, illustrated in Figure 1, comprises three steps. First, a *seed set* is defined by genes/proteins that share specific characteristics of interest which will be later used for growing a functional interaction network. This can be a set of proteins associated with a certain disease, involved in specific pathways, sharing other biological properties or transcripts that are differentially expressed in a condition of interest. In the second step, candidate proteins are extracted based on their functional similarity to the seed set and a *domain-specific similarity network* is generated by extending this set by all functionally related proteins. The notion of similarity is not necessarily restricted to functional annotation or interaction data but rather can cover any kind of genomic data, such as expression data, SNPs, sequences and phenotypes. Finally, in the last step *network centrality analysis* is performed to rank those proteins with respect to their relative importance within the network. The most central ones are presumed to be of functional importance for the specific network. Note that for simplicity we only referred to proteins in the description of the framework. However, our method is not restricted to proteins but is also applicable to genes depending on the biological question.

Translating the general framework into the context of identifying surface membrane factors interacting with HIV-1 implies that proteins, which are related to known HIV receptors through functional similarity or interaction with the same ligand(s), tend to be part of the same pathway and often share the same biological function. Therefore, if a network is built based on documented surface membrane factors that is extended with related genes, yet undiscovered surface proteins should also be central in the resulting network. To study this, we build an enriched HIV receptor network from known HIV receptors and rank all its proteins according to their centrality within the network. Highly ranked proteins are further analyzed to identify potentially novel surface membrane factors.

Below we explain the details of our method for identifying surface membrane factors interacting with HIV-1. One should keep in mind that the framework is neither domain nor disease specific and can be applied for various biological questions other than the one presented in this study.

### Data

We use a set of known HIV receptors, their functional annotations and human protein interaction data as a scaffold for building an HIV receptor network. The initial list is compiled by mining the literature and the ‘HIV-1, Human Protein Interaction Database’ [20]. A receptor is included if it is reported by at least two independent studies. This applies to 16 HIV receptors. However, three of them, namely Rdc1, Gpr15 and ChemR23, are not documented in the data gathered from protein interaction databases (see below) and thus have not been used in this study. Table 1 shows the final list of 13 HIV receptors including protein domain information (InterPro), literature references and their role in HIV infection. The list covers established receptors such as CD4 and DC-SIGN, HIV co-receptors CCR5 and CXCR4 as well as alternative co-receptors CCR2 and CCR3. Only recently reported co-receptors, such as XCR1 [21], have not yet been included since they were not documented by the time the study was conducted. However, we use a list of cell surface proteins that are reported to interact with HIV in a broad sense. Therefore, we do not limit our prediction method to receptors that only permit the entry of HIV into the primary cells.

**Table 1:** Initial set of HIV seed receptors.

Receptor	Receptor type	InterPro domains
**Ig-like and Other**		
CD4	Primary receptor for HIV	Ag 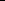 CD4, CD4-extracel, Ig-like, Ig-like 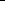 fold, Ig 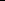 C2-set, Ig 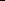 sub, Ig 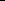 V-set 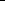 sub
**7-TM GPCR and CCR** 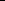 **rcpt**		
CCR5	Co-receptor with CD4	7TM 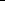 GPCR 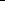 Rhodpsn, CC 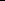 5 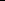 rcpt
CCR3	Alternative co-receptor with CD4	7TM 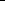 GPCR 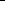 Rhodpsn, CC 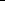 3 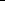 rcpt
CCR2	Alternative co-receptor with CD4	7TM 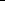 GPCR 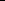 Rhodpsn, CC 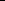 2 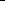 rcpt, CC 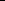 5 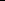 rcpt
CCR8	Alternative co-receptor with CD4	7TM 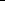 GPCR 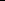 Rhodpsn, CC 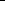 8 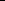 rcpt
CCR9	Alternative co-receptor with CD4	7TM 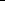 GPCR 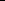 Rhodpsn, CC 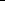 9 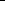 rcpt
CXCR4	Alternative co-receptor with CD4	7TM 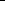 GPCR 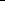 Rhodpsn, CXC 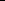 4 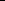 rcpt
CXCR6	Co-receptor	7TM 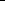 GPCR 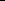 Rhodpsn, CXC 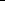 6 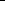 rcpt
CX3CR1	Co-receptor with CD4	7TM 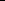 GPCR 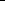 Rhodpsn, CX3C 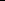 fract 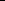 rcpt
**7-TM GPCR and Other**		
APJ	Alternative co-receptor	7TM 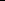 GPCR 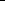 Rhodpsn, APJ 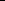 rcpt
GPR1	Alternative co-receptor	7TM 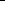 GPCR 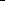 Rhodpsn, GPR1 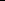 rcpt
**Integrin-** 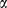		
ITGA4	Co-receptor with CD4	Int 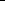 alpha 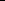 beta-p, Integrin 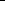 alpha, Integrin 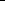 alpha-2, Integrin 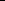 alpha 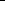 C
**C-type lectin and Other**		
DC-SIGN	Receptor for HIV	AntifreezeII, C-type 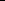 lectin

List of seed HIV receptors, including the receptor type and their functional domains. Receptors are grouped according to their functional domains (see Figure 5(a) for the distribution of those domains). A full table including the complete list of references that indicate the association to HIV is provided in Table S5.

Human protein interactions were obtained from the major public protein-protein interaction databases: DIP [22], IntAct [23], BIND [24], Mammalian MIPS [25], HPRD [26], MINT [27] and BioGRID [28]. From each database we retrieved the complete set of available human protein interactions. Table S1 provides the number of protein interactions obtained from each database by the time of this study. We integrated the different data sets by mapping the interacting proteins to unique protein identifiers from UniProt [29] or EntrezGene [30] and thus generating one comprehensive protein interaction map for our study. The integrated protein interaction set comprises 13,494 human proteins and 43,637 unique interactions observed between these proteins. Each protein included in the interaction map is associated with its respective protein domain information [31] and functional Gene Ontology (GO) annotations [32] (also retrieved from UniProt and EntrezGene).

### HIV receptor network

We generate a specific HIV receptor network using known receptors as seeds (see Table 1). We map each seed gene to its protein(s) thus growing a network around them [33]. The network is extended by adding proteins that either directly interact with any seed or that are functionally similar to at least one seed. Functional similarity between two proteins is determined by using a semantic similarity measure proposed by Couto *et al.* [34]. The formal definition of functional similarity is provided in the Text S1. In principle, proteins are considered as functionally similar if their semantic similarity to a seed protein is above the threshold of 0.7 (averaged across the three GO subontologies: molecular function, biological process and cellular component). Thereby, we only consider close and significant biological relationships.

Functionally related proteins are integrated into the network through weighted edges to the seeds. Edge weights are assigned by combining a protein interaction and a GO score. The protein interaction score is either 1 if an interaction is documented between a protein and a seed, and 0 otherwise. The GO score ranges between 0 and 1 (see Text S1) depending on the similarity of the GO annotations between two proteins, whereby 1 indicates functional equality and 0 indicates maximal functional distance. Interactions and functional similarities among all non-seed proteins are also included into the network.

We exploit protein interaction because it strengthens the relationship between (similar) receptors interacting with the same ligand. Human interaction data, however, is still incomplete and will not cover the functional space for our analysis. Therefore, we also integrate functional data to capture cellular surface proteins that show significant functional similarity with the seed receptors. Nevertheless, the functional coverage is still limited and currently only a fraction of the genome is annotated with pathways, functions and phenotypes [19]. Hence, we integrate predicted functions in our framework to functionally enrich proteins that are weakly or not annotated at all.

### Functional enrichment

To functionally enrich the HIV network we apply a network-based function prediction method to derive additional annotations. This method compares protein interaction networks across multiple species to detect evolutionarily and functionally conserved subgraphs. This involves the identification of orthologous proteins (using OrthoMCL [35]) and the detection and assembly of conserved interactions. Within each conserved subgraph we infer novel protein functions from orthology relationships across species and along conserved interactions of neighboring proteins within a species (Jaeger *et al.* submitted, see [36] for early work). Predicted functions are added to the set of confirmed functions to better characterize proteins that are weakly or not annotated at all. The functional enrichment increases the final cross-validation recovery rate up to 30% (see Text S1, Table S2 and Figures S1 and S2 for detailed comparisons).

### HIV network centrality analysis

Network centrality analysis is particularly useful for identifying key elements in different biological processes. In general, networks are modeled as mathematical objects called graphs. A graph is an abstract presentation of a set of objects that are connected by links. In the most common sense a graph 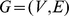 consists of a finite set of vertices 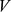 and edges 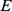 whereas an edge 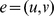 connects two vertices 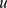 and 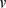. Centrality, on the other hand, is formally defined as a function 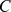 that determines a numerical value 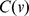 for every vertex 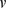 in a graph. We are interested in the ranking of vertices of the given graph 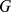, thus we follow the convention that a vertex 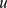 is more important than another vertex 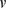 if and only if 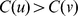 [37].

Different centrality measures have been proposed for analyzing various types of biological networks [37]. Established measures are degree centrality, closeness centrality, betweenness centrality and PageRank centrality. Here, we chose PageRank [38] to identify the most important factors within the HIV receptor network since the PageRank algorithm assigns numerical scores to each node to determine its relative importance within the network based on the assumption that not all relationships are equally important for determining the centrality of a node. Thus, links to high-scoring nodes contribute more to the PageRank centrality of a node than links to low-scoring nodes.

We used the PageRank centrality measure to discover novel surface membrane factors that are involved in HIV-1 infection. Accordingly, we rank all proteins with respect to their PageRank centrality within the network using the igraph library in R [39]. Clearly, we expect the seed receptors to be highly ranked in the ordered list, since our construction algorithm naturally places them in a central position. Nevertheless, not all seed receptors are central, and many non-seed proteins are ranked high. We are especially interested in the latter since these are promising candidates for novel surface membrane factors. An appropriate ranking is essential for deciding which factors should be investigated further, e.g. in follow-up experiments.

### Validation

We validate our method and the results as follows: First, we use leave-one-out cross-validation to assess the predictive power for finding novel surface membrane HIV factors. Second, we determine the statistical significance of our results by comparing them to a random control set. For cross-validation we remove one seed receptor from the initial list and try to re-discover this receptor using our method. We build an HIV receptor network from the remaining receptors and rank the proteins according to their centrality within the network. Subsequently, we determine whether the left-out receptor is re-discovered and at which position of the ranked list. We repeat this procedure for each seed receptor and determine the average recovery rate across all receptors.

To determine the statistical significance of the results, we compare them to two random control sets. The first set, 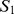, comprises all proteins from the human interaction network as candidates resulting in 13,494 proteins. The second set, 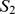, is stricter and contains only proteins with receptor properties, simulating a more informed manual search. To generate this set we use specific GO annotations that imply a receptor activity (see Table S3) since there is no general receptor definition indicating whether a protein is a receptor or not. Thus, 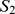 is formed by filtering proteins from the interaction data that are annotated with at least one of these specific GO terms. This results in 2,512 candidates - covering 12 out of 13 seed receptors (ITGA4 is missing due to insufficient functional annotation). We randomly draw 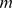 samples from each control set, where 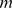 corresponds to the average number of proteins within the HIV network and determine whether the known receptors are among the samples. This is repeated 1,000 times and an average recovery rate is calculated which is later compared to the recovery rate from our ranking method.

## Results

We have designed a framework for discovering novel surface membrane factors interacting with HIV-1. To this end, we use protein interaction, protein function, and network centrality analysis to determine yet uncharacterized surface membrane proteins based on their functional similarity and topological closeness to receptors that are known to interact with HIV.

**Figure 2:**
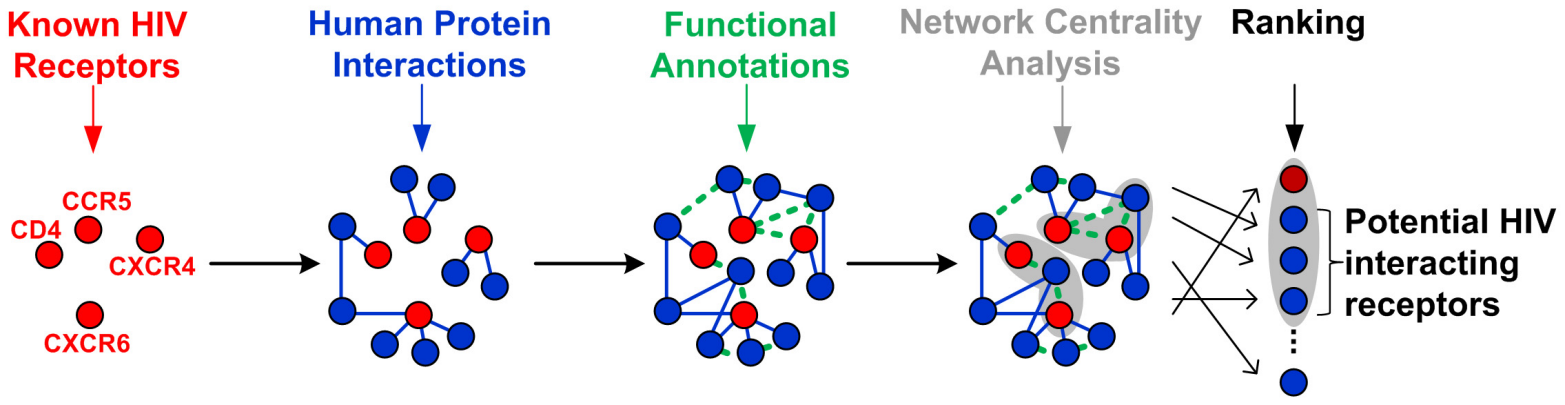
Illustration of the key steps in the prediction method. Starting from the seed HIV receptors we add proteins that 1) have direct interaction (blue solid edges) or 2) are functionally similar (green dashed edges) to the known receptors to generate an enriched HIV receptor network. Proteins are ranked according to their centrality within the receptor network. Proteins in shaded areas represent highly central proteins.

Our strategy is based on the assumption that proteins, which are related to known HIV receptors through functional similarity or direct interaction with the same ligands(s), tend to be part of the same pathway and often share the same biological function. Therefore, an enriched HIV receptor network is built from documented surface membrane factors by populating it with functionally related proteins that either interact directly with or show significant functional similarity to any known factor. Subsequently, all proteins are ranked according to their centrality within the network. The underlying principle of the centrality analysis presumes that the most central proteins in a domain-specific network are likely to be of high functional relevance [40]. Thus, yet undiscovered but prospective surface proteins should also be central in the network. Highly ranked proteins are analyzed further to identify potentially novel surface membrane factors. The key steps of our inference method are illustrated in Figure 2.

In the following subsections, we first evaluate the performance of the prediction method. Subsequently, we investigate the most promising predictions by exploring literature on their functional domains, expression levels and reported clinical evidence.

### Cross-validation

Cross-validation is performed on 13 known HIV receptors to evaluate the predictive power of the method. Overall, we achieved a re-discovery rate of 92% (12 out of 13). ITGA4 was not re-discovered by our method, due to its insufficient annotation and low functional similarity to the other 12 receptors.

We studied the recovery rates using interaction data and GO annotation with and without functional enrichment. The comparison shows that the total number of re-discovered receptors is significantly higher when functionally enriched data is employed (see Text S1, Table S2 and Figure S1 and S2 for detailed results). Consequently, interaction data in combination with enriched functional annotation are chosen for further analysis.

The same evaluation was performed using random control sets, 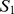 and 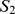. The random recovery rates are compared to the network-driven recovery rate to assess the statistical significance. We determine the fraction of seed receptors that can be discovered when randomly sampling from the complete protein set (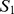) and a subset including only surface membrane proteins (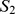) (see Methods). On average, we discover 0.69 and 3.4 of the 13 seed receptors when sampling from 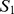 and 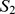, respectively, which results in random recovery rates of 5.3% and 26.2%. The comparison of recovery rates shows that the network-driven recovery rate of 92% is clearly superior to the random recovery rates of 5.3% and 26.2%. The t-test confirms that the observed superiority over the control sets is statistically highly significant (p-value
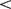
2.2e-16) and thus underlines the advantage of our network-driven strategy over the random approach.

**Figure 3:**
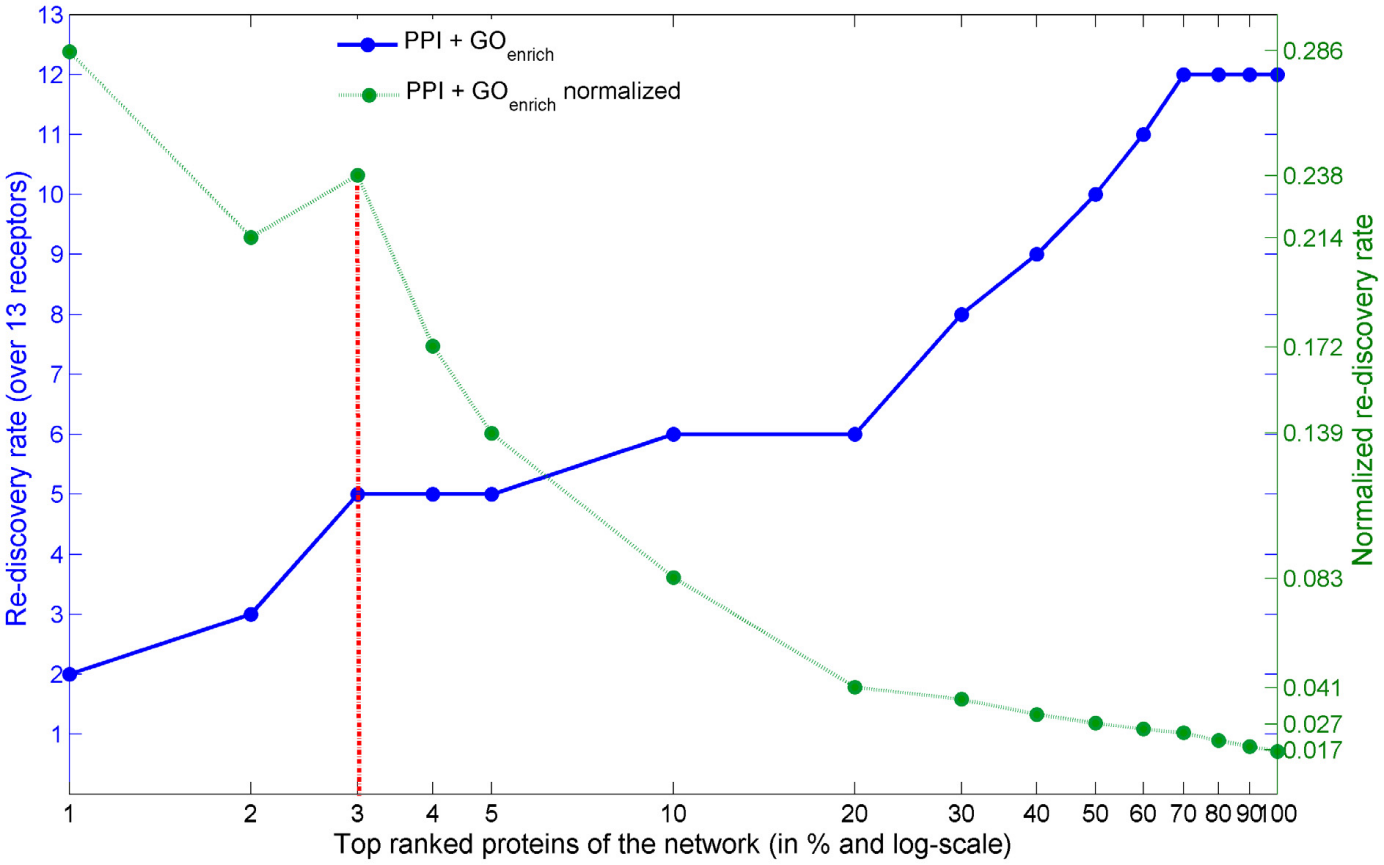
Results of the leave-one-out cross-validation over the 13 seed receptors. The average receptor re-discovery rate is determined for the different ranks (in %) of the HIV network. The original re-discovery rate (left y-axis and solid line) is compared to the normalized re-discovery rate (right y-axis and dashed line). The red dashed line indicates the chosen cut-off. The x-axis is in log-scale to focus on the highest ranks (1% to 5%).

For the prediction of novel surface membrane factors, we investigate the trade-off between discovering potential candidates vs. false positives by normalizing the recovery rate by the number of proteins considered at each rank. Figure 3 compares original and normalized recovery rates across the prioritized protein list. The receptor-per-protein ratio is used to assess the probability to identify new HIV interacting surface proteins. The most significant discovery ratio is 29% (2/7) considering the top 1% proteins. The second best discovery ratio is achieved at 3%, where the probability of rediscovering a known surface membrane factor is 24% (5/21). Note that the probabilities are estimated from the cross-validation on known data and therefore provide *lower bounds* since all novel findings are counted as false positives.

We used these receptor-per-protein ratios to define a cut-off to select candidates from the prioritized list. We choose 3% as threshold, since it presents a sensible trade-off between potential candidates and false positives (see above) while yielding a reasonable number of novel candidates. Thus, the top 21 proteins in the ranked list are considered as surface membrane factor candidates.

### Predicting novel HIV surface membrane factors

Finally, we consider all 13 known HIV receptors as seeds to build an HIV receptor network with 739 proteins (726 candidates) and 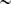
80,000 functional relationships (note that during cross-validation we always removed one of the seeds). We run the PageRank algorithm and obtained a list of centrality-ranked proteins. Seed receptors are removed from the list since they are (by definition) highly ranked. We apply the chosen threshold and consider the first 21 proteins as host factor candidates. Table 2 presents the top-ranked candidates including their InterPro domains and cell types.

**Table 2:** List of inferred surface membrane factors.

Receptor	Receptor-specific InterPro domains	Cell types	Association with HIV
**7-TM GPCR and Other**			
HTR6	Not applicable	Uniform expression^1^	+
HTR1B	5HT1B 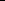 rcpt	Uniform expression^1^	?
HTR1E	5HT1F 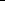 rcpt	Uniform expression^1^	?
RXFP2	LDL 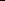 rcpt 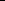 classA 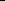 cys-rich 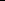 rpt, Leu-rich 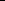 rpt, LRR-contain 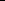 N, Leu-rich 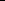 rpt 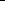 typical-subtyp, Relaxin 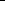 rcpt	Low expression	?
RXFP1	LDL 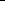 rcpt 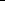 classA 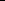 cys-rich, Leu-rich 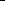 rpt, LRR-contain 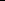 N, Leu-rich 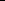 rpt 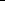 typical-subtyp, Relaxin 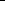 rcpt	No expression profiles available	?
GPR17	P2 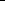 purnocptor	Uniform expression^1^	?
GPR182	G10D 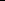 rcpt	Uniform expression^1^	?
NPBWR2	Neuropept 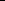 W 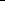 rcpt	Uniform expression^1^	−
**7-TM GPCR and CCR** 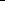 **rcpt**			
CCR1	CC 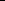 1 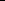 rcpt	High expression: whole blood, monocytes, myeloid, dendritic cell	+
CCBP2	CXC 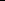 4 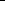 rcpt	Uniform expression^1^	+/−
**7-TM GPCR**			
DARC	Duffy 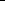 cmk 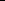 rcpt	High expression: (early) erythroid, endothelial cells	+
**Ig-like and Other**			
CD2	Ag 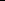 CD2, Ig-like 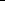 fold, Ig 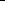 C2-set, Ig 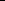 V-set, T-cell 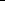 sdhesion 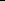 molc 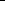 CD2	High expression: dendritic, myeloid, monocytes, NK, CD8 and CD4 T cells, whole blood	+
CSF3R	FN 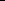 III, Hematopoietin 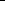 rcpt 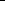 gp130 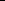 CS, IgC2-like 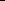 lig-bd	High expression: myeloid cells, monocytes and whole blood	+
IL1R1	Ig, Ig-like 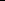 fold, Ig 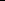 sub, IL1 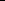 rcpt 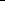 1, IL1R 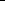 rcpt	No expression profile available	−
CD79B	Ig-like 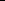 fold, Ig 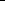 sub, Ig 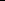 V-set, Phos 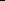 immunorcpt 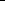 sig 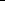 ITAM	High expression: CD34, endothelial and dendritic cells	+
IL6ST	FN 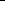 III, Hematopoietin 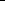 rcpt 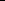 gp130 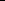 CS, Ig-like 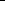 fold, IgC2-like 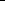 lig-bd	Uniform expression^1^	+
**TNFR** 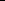 **Cys** 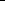 **rich** 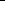 **reg and Other**			
TNFRSF5	Fas 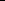 rcpt	High expression: B lymphoblasts	+
TNFRSF3	TNFR 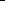 3 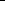 LTBR	High expression: myeloid, monocytes and whole blood	+
**Other**			
CD97	EGF-type 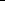 Asp/Asn 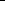 hydroxyl 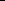 site, EGF 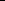 Ca 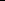 bd 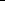 2, GPCR 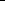 2 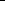 CD97, GPCR 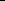 2 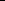 secretin-like, GPS 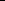 dom	High expression: CD34, B lymphoblast, dendritic cells, CD8 and CD4 T-cells, NK, myeloid, monocytes	+
GP1BB	LRR-contain 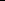 N, Cys-rich 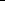 flank 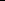 reg 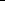 C	High expression: CD34, monocytes and whole blood	?
GYPB	Glycophorin	High expression: (early) erythroid and endothelial cells	?

List of the potential surface membrane proteins that result from our method, including functional domains and cell types. Predictions that are associated with HIV in earlier studies are marked with ‘+’. ‘−’ indicates predictions with negative evidence. For predictions without literature on interaction the association remains unclear (shown by ‘?’). The list of supporting references is provided in Table S6.

1 Uniform expression in CD34, endothelial, B lymphoblasts, dendritic, myeloid, monocytes, NK, CD8 and CD4 T cells, and whole blood.

**Figure 4:**
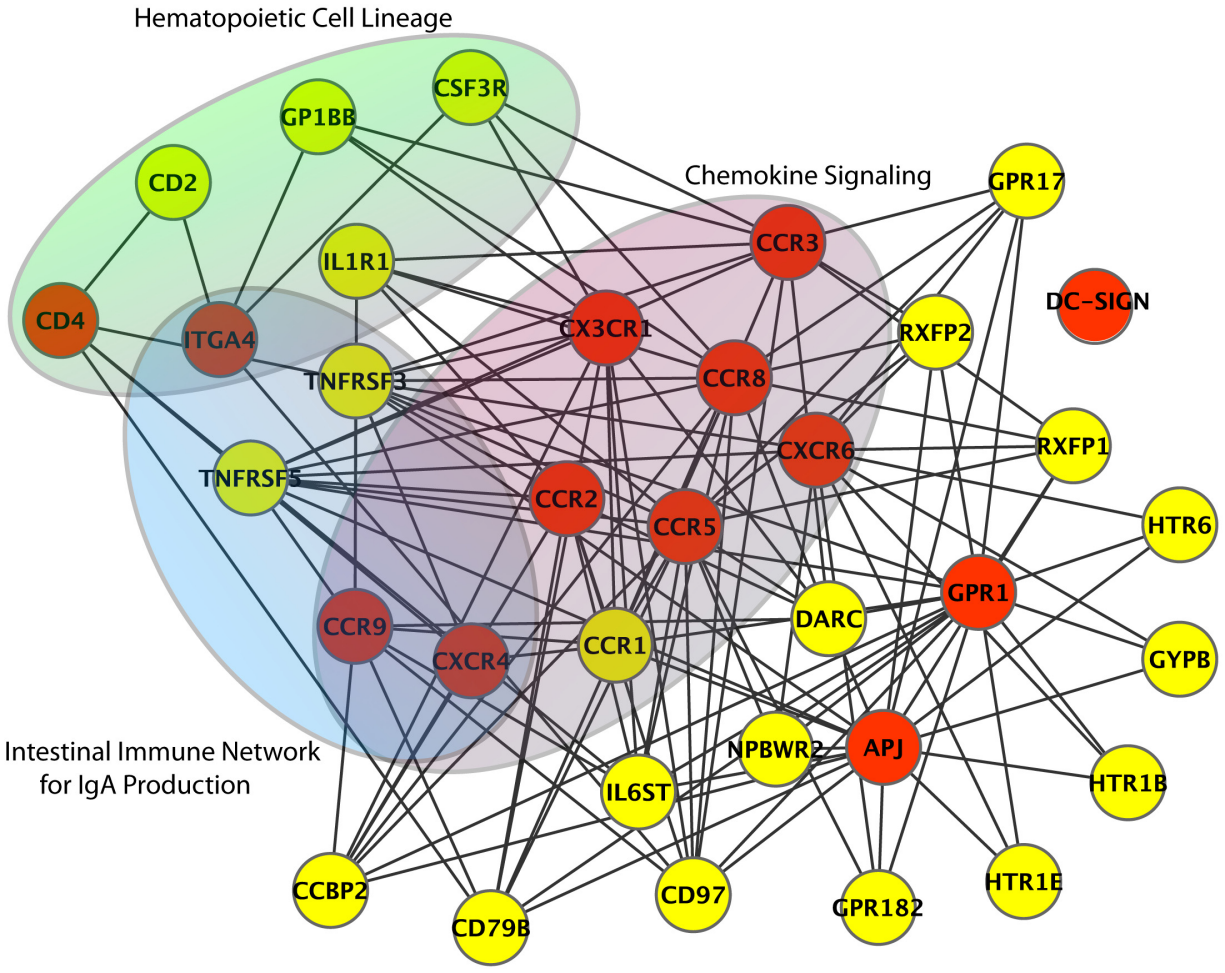
Subnetwork of the generated HIV receptor network. The subnetwork focuses on the functional relationships between the seed receptors (red) and the predicted surface membrane proteins (yellow) within the HIV receptor network. Non-seeds and non-candidate proteins are not shown confusion. Significantly enriched pathways within this subnetwork are additionally highlighted.

Figure 4 shows the subnetwork from the full HIV receptor network that exhibits only the direct functional relationships between seed receptors and predicted surface membrane factors. The analysis of the known and predicted surface membrane factors regarding their annotated KEGG pathways [41] revealed the involvement of three pathways, namely the chemokine signaling pathway (hsa04062), the hematopoietic cell lineage (hsa04640) and the intestinal immune network for IgA production (hsa04672).

### Support for predictions

We assess the relevancy of the candidates using evidence that supports an association with HIV. We investigate the predictions with respect to functional domains, cell types, expression levels, associated SNPs and chromosomal locations.

#### Receptor domains

We analyze our predictions by comparing their functional protein domains to the domains of the known seed receptors assuming that overlapping functional domains indicate similar protein properties, e.g. binding the same ligand, and functional similarity [42]. Common protein domains of the seed receptors are:

1. G-protein-coupled receptors (GPCR) rhodopsin-like superfamily and 7 transmembrane (7-TM) GPCR rhodopsin-like domains (7-TM GPCR)
2. Chemokine receptor domains (CCR
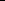
rcpt)
3. Immunoglobulin and related domains (Ig-like)
4. C-type lectin and related domains (C-type lectin like)
5. Integrin alpha and related domains (Integrin alpha)

**Figure 5:**
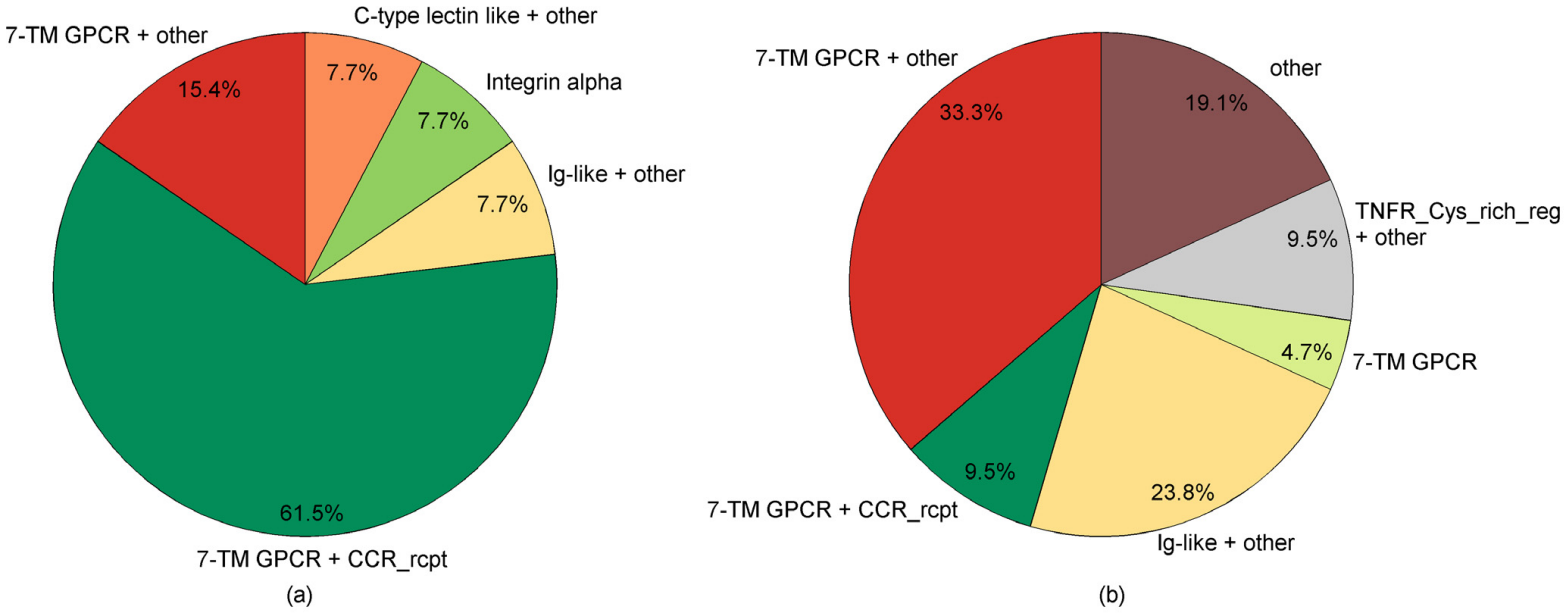
Overview on the distribution of protein domains. Distribution of the protein domains for (a) seed receptors and (b) predicted surface membrane factors.

The distribution of the domains among the seed receptors is shown in Figure 5(a).

Predicted surface membrane factors are grouped according to their functional domains (see Table 2) which results in GPCR with chemokine domains, GPCR without chemokine domains, Ig-like receptors and receptors without any overlapping domains. The respective domain distribution is displayed in Figure 5(b). The largest domain overlap is found for 7-TM GPCR rhodopsin-like domains. Half of the predictions have this particular domain, which is also overrepresented in the set of seed receptors (10 of 13, see Figure 5(a)). In addition, CCR1 and CCBP2 share a chemokine domain, which is very frequent in the set of initial receptors (8 of 13). Moreover, five predicted surface membrane factors have Ig-like domains that match the primary HIV receptor CD4.

The amount of overlapping functional domains indicates that the functional characteristics of the initial HIV binding receptors are reflected in predicted surface factors. In particular, GPCRs have a broad usage spectrum as co-receptors by primary isolates of HIV [21] and specifically chemokine receptors are known as co-receptors for HIV [43]. Strikingly, CCR1 and CCBP2 share both 7-TM GPCR rhodopsin-like and chemokine domains and are reported as co-receptors of HIV. However, receptors without any overlapping domains might present unprecedented characteristics that are not documented in the initial set but are reflected in their complementary domain diversity (see Figure 5(b)).

#### Chromosomal locations

Genes with similar properties are sometimes located in the same regions of the human genome. Thus, the genomic location of a gene is often taken into account when new candidate genes are associated with a disease. The reason is that mapping those candidates to a region containing other genes associated to the same disease further supports the association. For example, HIV binding human CC chemokine receptor genes are known to cluster within the 3p21.3 region of the genome [44].

We determine the chromosomal location of the predicted surface proteins and study whether they cluster together with other candidates or known seed factors. The chromosomal location for each seed and prediction retrieved from EntrezGene is shown in Table S4. When considering the known receptors there is a group of six chemokine receptors that map to the CCR cluster within 3p21.3, and also two receptors, CCR1 and CCBP2, from the predicted set are associated to this region. However, the remaining ones are located on different chromosomes and do not map together. Only CD97 and DC-SIGN, and GPR17 and CXCR4 are mapped together to 19p13 and 2q21, respectively.

## Discussion

The involvement of co-receptors and surface membrane proteins assisting HIV-1 infection and contributing to viral pathogenesis always has been underestimated [21]. Only a limited number of studies aim to elucidate the role of surface membrane factors interacting with viral proteins even though they are potential amenable drug targets for HIV therapeutics [45], [46].

We predict 21 surface membrane HIV factors that are potentially involved in the different stages of infection influencing the progression of the disease. Remarkably, among these cell surface proteins, three have confirmed functions in HIV infection, seven have been reported by at least two other studies and eleven predictions are novel findings that deserve experimental investigation. It is important to note that the high success rate of our method, as shown using cross-validation, strongly implicates that our predictions can be the missing piece of the puzzle.

### Experimentally confirmed predictions

#### CCR1

The C-C chemokine receptor type 1 is a GPCR that mediates signal transduction and the recruitment of effector immune cells to inflammation sites. It is highly expressed in immune system cells, such as myeloids, monocytes, dendritic cells and whole blood. Independent studies confirmed the usage of CCR1 along with CD4 for the entry of HIV into target cells [21], [47].

#### CCBP2

The Chemokine-binding protein 2 is another chemokine receptor that is documented to function as alternative co-receptor for HIV [48].

#### DARC

The Duffy antigen/chemokine receptor belongs to the family of erythrocyte chemokine receptors that bind chemokines. It is highly expressed on red blood cells (RBCs). Several studies demonstrated the binding of HIV-1 to RBCs through DARC enabling RBCs to transmit HIV to peripheral blood mononuclear cells. However, binding HIV to DARC does not permit viral entry but retains the virus viability and mediates trans-infection of HIV-1 from RBCs to susceptible T cells [49], [50]. Recently, He *et al.* reported that the DARC −46C/C genotype is associated with an increase of 40% in the odds of acquiring HIV-1 in African Americans [50]. However, follow-up studies on different cohorts [51]–[53] or with correction for population stratification [54] could not establish a significant association of this DARC polymorphism and the increased risk for HIV-1 acquisition or disease progression. Although DARC's association with HIV has been established some questions remain regarding its influence on HIV-1 acquisition and progression.

### Prediction with direct and indirect experimental support

#### CD97

It belongs to the EGF-TM7 family of class II 7-TM molecules and is present on the surface of most activated leukocytes. It is broadly expressed on most hematopetic cells, activated lymphocytes, macrophages, dendritic cells, granulocytes, monocytes and undergoes a rapid up-regulation during T and B cells activation. Recently, CD97 was identified in a large-scale genome RNAi screening as one of six uncharacterized host factors that are required for HIV replication [45] suggesting its crucial post-integration role. Furthermore, Kop *et al.* [55] showed that CD97 is present on the surface of all human lymphocytes in blood and lymphoid tissue and confirm its up-regulation upon cellular activation. In addition, they demonstrated significant differences in the expression levels between lymphocytes. For instance, T and NK cells possess higher levels of CD97 than B cells and memory CD4+ (but not CD8+) T cells express more CD97 than naïve cells. These differences might present the missing factor that is required for active infection of naïve T cells in early infection because CD97 is highly expressed in activated memory CD4+ cells but not in naïve subsets. To confirm this hypothesis longitudinal testing of in-vivo expression of CD97 is necessary in patients going through co-receptor switch.

#### CSF3R

The granulocyte colony-stimulating factor receptor is the receptor for colony stimulating factor 3 (G-CSF), a cytokine that controls the production, differentiation, and function of granulocytes. CSF3R is highly expressed on monocytes and activated T cells [56]. Its ligand modulates cytokine production in monocytes and lymphocytes. In particular, CSF3R is thought to play a role after viral DNA synthesis. The indirect influence on infection and replication in human cells has been demonstrated through the binding of recombinant G-CSF (rG-CSF). rG-CSF is able to activate replication of HIV-1 during hematopoietic stem cell mobilization in HIV-1 infected persons [57] and stimulates viral production through binding to CSF3R that is expressed on HIV-1 chronically infected cell lines [58]. The direct impact of CSF3R on HIV replication has been documented recently [46]. Besides, CSF3R has been linked to the developing congenital neutropenia [59]. This is particularly interesting since DARC has also been associated with benign ethnic neutropenia observed in people with African descent [60]. Thus, we hypothesize that in addition to the genetic predisposition of DARC, CSF3R can account for the observed differences in HIV induced neutropenia.

#### TNFRSF3

Also known as Lymphotoxin-beta receptor (LT-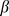
R), is a member of the tumor necrosis factor (TNF) receptor superfamily that participates in the regulation of immune and inflammatory responses by propagating signals that regulate cell survival or death through activation of NF-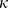
B [61]. LT-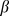
R is expressed on myeloids, dendritic cells and monocytes, which play a critical role in the progression towards AIDS by providing a major source and reservoir of virus when the T cell population is depleted [12], [62]. Signaling through LT-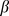
R via its ligand LT-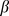 stimulates viral replication within infected monocytes [63].

#### TNFRSF5 (CD40)

CD40 is a type I membrane glycoprotein of TNF receptor superfamily and is expressed on B-lymphocytes. Its ligand CD40L is expressed mainly in activated CD4+ T lymphocytes. The interaction between CD40 and CD40L leads to the activation and differentiation of B-lymphocytes [64]. This mechanism constitutes a non-redundant central role in humoral and cell-mediated immunity. Early studies identified a link between CD40L expression and progression to AIDS [65]. Recently it has been demonstrated that HIV-1 promotes CD4+ T cell infection by inserting CD40L into emerging viral particles and trans-activating B cells in a CD40 dependent manner [66].

#### CD2

It is typically expressed on T cells and most CD3- Natural Killer (NK) cells. It mediates intracellular adhesion in T lymphocytes and targets cells for lysis in NK cells. CD2 has a pivotal role in activating and inducing latent HIV-1 replication in resting CD4+ T cells through the CD2 pathway [67]. The CD2 pathway is also reported to increase HIV production in-vivo [68]. Moreover, a longitudinal study on ‘Highly active antiretroviral therapy’ over a three-year period showed a significant increase of CD2 expression on peripheral blood mononuclear cells as well as a slight increase in viral load over the same period [69].

#### IL6ST (GP130)

The Glycoprotein 130 is a transmembrane protein that controls the activity of cytokines, such as IL- 6, IL-11, IL-27 and leukemia inhibitory factor (LIF) [70]. It is expressed in many tissues ranging from gut epithelia to astrocytes and T cell subsets. GP130 was associated with HIV when studying LIF's protective role against vertical transmission of HIV-1 from mother to child [71]. Both are significantly up-regulated in lymphoid tissue [72] and found in high concentrations in plasma samples of patients [73] during primary HIV-1 infection.

Moreover, GP130 is involved in differentiation among T-helper cell (Th) subsets. A lack of GP130 in T cell specific conditional gp130 deficient mice models causes the activation of Th2 and regulatory T cell pathways [70]. In the case of HIV infection, this change in T cell differentiation dynamics may be responsible for various levels of disease progression observed in different individuals. The imbalance of Th subsets is also a strong predictor of pathogenic SIV infection in primate models [74]. Similarly, successful CD4+ T cell restoration was associated with enhanced Th17 CD4+ T cell accumulation when comparing gut associated lymphoid tissue recovery rates from HIV infected individuals [75], [76].

#### CD79B (B29, IGB)

CD79 is a transmembrane protein that forms a complex with the B-cell receptor (BCR) and generates a signal following recognition of an antigen by the BCR. It is expressed almost exclusively on B cells and B-cells neoplasms [76]. It is composed of two distinct chains called CD79A and CD79B. CD79B plays an important role in BCR expression in B cell development [77]. HIV Gp120 is documented to down-regulate CD79B [78] but its underlying mechanism is not yet understood. In theory, down-regulation of CD79B leads to reduced capacity of B-cells to bind antigens and more importantly to a decrease in HIV specific antibody formation [79].

We are aware of the difficulties for implicating HIV-1 strains efficiently using alternative co-receptors for infection of transfected cells. Experimental testing usually requires coculturing of virus strains showing broad co-receptor usage [13], [21], [80] with appropriate transfected cell lines. However, we believe that this effort is necessary for unraveling potential causes underlying confounding traits of HIV-1 infection.

### Conclusions

We use a systems biology framework that integrates protein interactions, functional annotation and protein domains for inferring surface membrane factors interacting with HIV. The analysis of our predictions confirms that surface membrane proteins, even though are targeted under different conditions, are likely to be part of the same functional pathways.

We infer ten surface proteins that are involved in a cascade of events in HIV infection. Their involvement ranges from serving as co-receptors for cell entry (CCR1 and CCBP2), mediating trans-infection (DARC), activating immune cells (CD97) to inducing viral production from latently infected cells (CSF3R, TNFRSF3 and CD2).

We also present eleven original predictions that are potential HIV interacting factors (see Table 2). In particular, the platelet glycoprotein Ib (GPIb) is a surface membrane protein of platelets. Mutations in the GPIb beta subunit are associated with Bernard-Soulier syndrome which is characterized by thrombocytopenia, circulating giant platelets, and prolonged bleeding time [81]. We speculate that the prolonged interaction of blood platelet expressed GP1BB with HIV might be responsible for thrombocytopenia observed in HIV infection. Furthermore, the relaxin receptors RXFP1 and RXFP2 are known to be expressed on the acrosome of elongated spermatids [82], [83]. Their intron rich gene organization indicates alternatively spliced variants. This suggests the existence of different protein isoforms that contribute to their diverse expression *in-vivo*. Their association with HIV might explain the different rates of evolution observed in seminal versus blood plasma of infected patients [84]. Moreover, either one or both receptors might be involved in viral hijacking of the spermatozoa in viral transmission [85].

Several seed receptors, such as CCR5, CCR2 and CX3CR1 [86], [87], have been associated with SNPs that contribute to different disease outcome. Among the 21 predicted factors, except for the controversial −46C/C in DARC, SNPs in CCR1, CCBP2, HTR6, HTR1B, HTR1E, CSF3R, IL1R1, TNFRSF5 are associated with one or more clinical phenotypes but their relation to HIV infection has not been investigated. Thus, we encourage investigating the SNPs from the predicted surface membrane factors for association with HIV to study their potential effect on HIV infection.

Throughout the manuscript we have presented a novel method and its application for identifying surface membrane factors for HIV-1. However, we emphasize that the presented framework is neither domain nor disease specific. More precisely, our approach is only depending on the initial (seed) data that is used to establish characteristic functional similarities. Thus, it can be employed for many biological questions other than the one discussed in this manuscript. Potential further applications include, for instance, clinical genetic studies for determining the downstream components of recently discovered disease genes, or drug-target testing for investigating possible effects/interactions of candidate compounds with proteins other than the intended targets.

Consequently, in this manuscript we present promising surface membrane factors that are potentially involved in HIV-1 infection. Testing these novel hypotheses with target-oriented *in-vivo* experiments is crucial to fully understand their impact on HIV-1 infection.

## Supporting Information

Figure S1 Comparison of the cross-validation results over the 13 seed receptors for the different HIV receptor network types with (i) interaction data only (PPI), (ii) interaction data and manual functional annotations (PPI+GO) and (iii) interaction data in combination with enriched functional annotation (manually curated and predicted function) (PPI+GO enrich).
(0.54 MB TIF)

Figure S2 Original rediscovery rates (left y-axis and solid lines) across the ranked list in comparison to the normalized rediscovery rates (right y-axis and dashed lines) for PPI+GO and PPI+GO enrich. The x-axis is in log-scale to focus on the higher resolution of the top ranks.
(0.15 MB TIF)

Table S1 The number of human protein interactions retrieved from each database by the time of our study.
(0.05 MB PDF)

Table S2 Average number of proteins (network size) comprised in each network and the number of seeds that are rediscovered during cross-validation when considering different data for generating the specific HIV receptor network.
(0.01 MB PDF)

Table S3 List of functional annotation from Gene Ontology that are used to filter for receptor proteins for the control sets.
(0.01 MB PDF)

Table S4 Chromosomal location of known and predicted surface membrane proteins. Similar chromosome regions are colored similarly.
(0.02 MB PDF)

Table S5 List of seed HIV receptors, including receptor name and type, their functional domains and references indicating an association with HIV-1 infection.
(0.07 MB PDF)

Table S6 List of the potential HIV surface membrane factors inferred by our method, including their functional domains, cell types and references assessing an association with HIV.
(0.20 MB PDF)

Text S1 Definition of functional similarity and description of the different HIV receptor network types generated from different functional data, including a detailed performance comparison of their ability to infer novel surface membrane factors interacting with HIV-1.
(0.06 MB PDF)
