# Supplementary figures and images for: Inference of Surface Membrane Factors of HIV-1 Infection through Functional Interaction Networks
Source: PLoS One. 2010 Oct 12;5(10):e13139. doi: 10.1371/journal.pone.0013139 (PMC2953485; doi:10.1371/journal.pone.0013139)

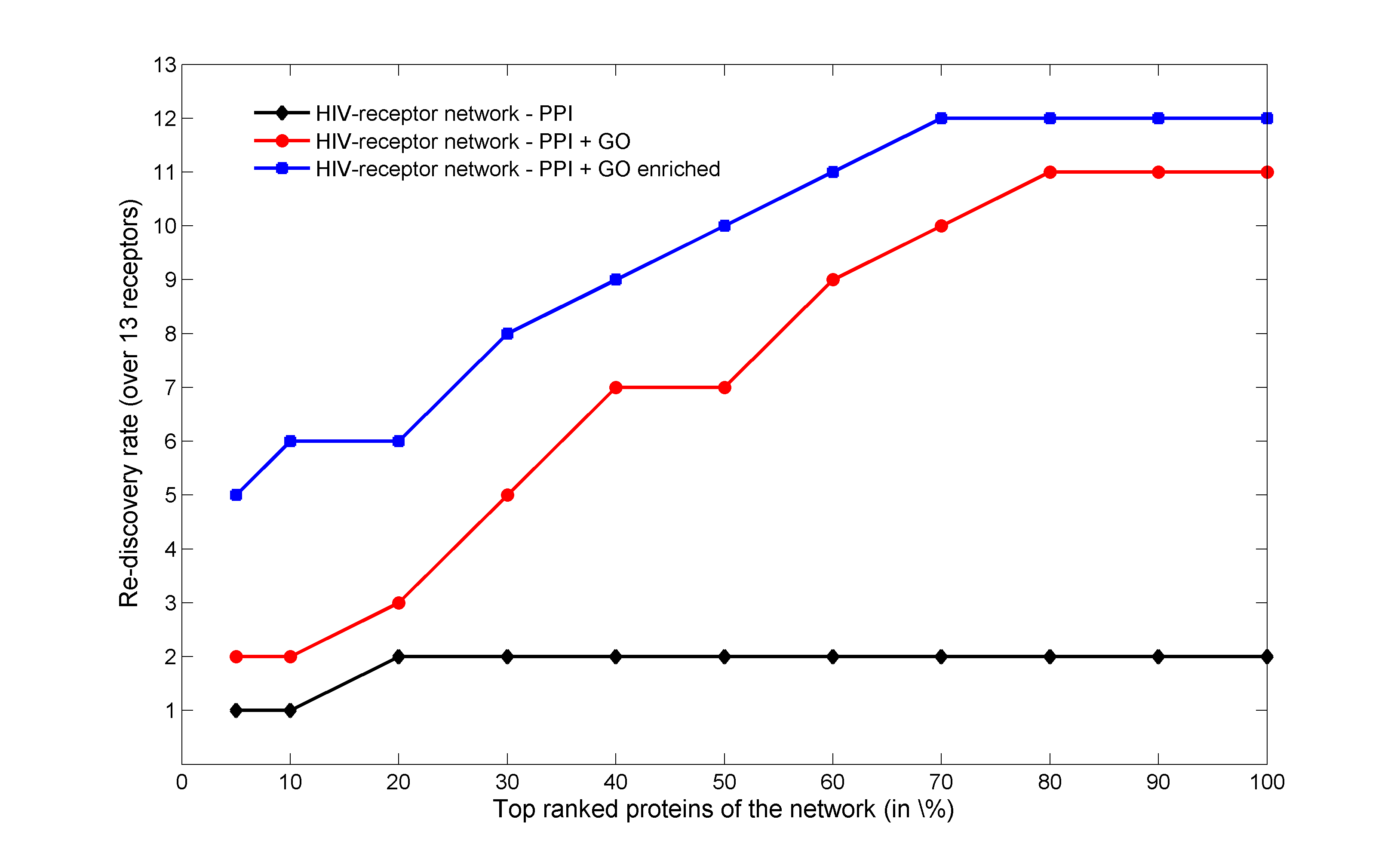

Supplement: Figure S1 — Comparison of the cross-validation results over the 13 seed receptors for the different HIV receptor network types with (i) interaction data only (PPI), (ii) interaction data and manual functional annotations (PPI+GO) and (iii) interaction data in combination with enriched functional annotation (manually curated and predicted function) (PPI+GO enrich). (0.54 MB TIF) [file pone.0013139.s001.tif]

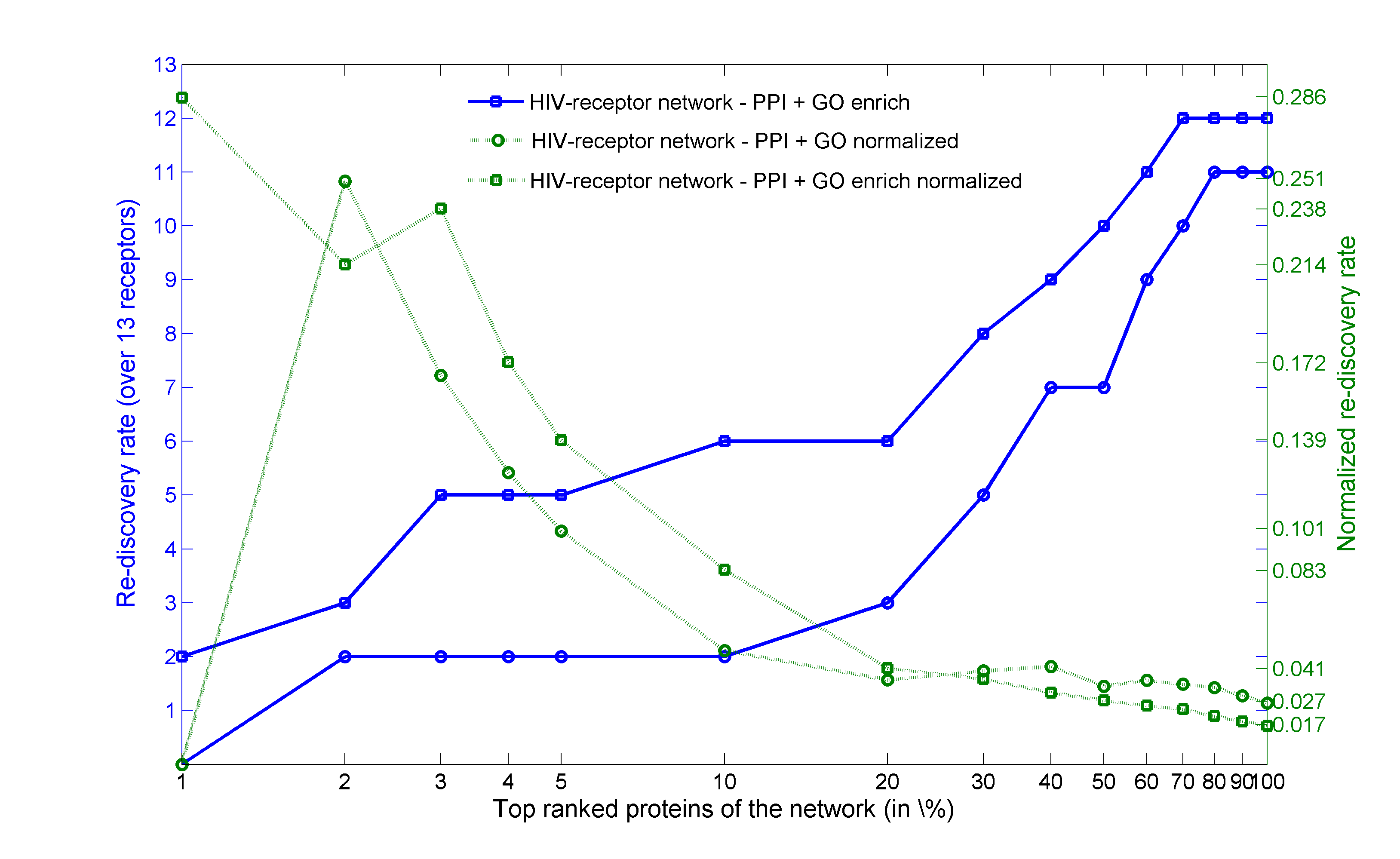

Supplement: Figure S2 — Original rediscovery rates (left y-axis and solid lines) across the ranked list in comparison to the normalized rediscovery rates (right y-axis and dashed lines) for PPI+GO and PPI+GO enrich. The x-axis is in log-scale to focus on the higher resolution of the top ranks. (0.15 MB TIF) [file pone.0013139.s002.tif]
