# Supplementary material for: Inference of Surface Membrane Factors of HIV-1 Infection through Functional Interaction Networks
Source: PLoS One. 2010 Oct 12;5(10):e13139. doi: 10.1371/journal.pone.0013139 (PMC2953485; doi:10.1371/journal.pone.0013139)
Supplement: Table S1 — The number of human protein interactions retrieved from each database by the time of our study. (0.05 MB PDF) [file pone.0013139.s003.pdf]

Table S1: The number of human protein interactions retrieved from each database by the time of our study. The integration of the protein interactions from the different databases results in a protein interaction set with 13,494 human proteins and 43,637 unique interactions between these proteins. Note, that there is an overlap between the databases, thus the numbers do not added up to the final number unique interactions.

PPI Database	Number of human protein interactions
**MIPS-MPPI**	127
**DIP**	3045
**MINT**	3160
**BIND**	5969
**BioGRID**	12779
**IntAct**	14298
**HPRD**	19215
