# Supplementary material for: Inference of Surface Membrane Factors of HIV-1 Infection through Functional Interaction Networks
Source: PLoS One. 2010 Oct 12;5(10):e13139. doi: 10.1371/journal.pone.0013139 (PMC2953485; doi:10.1371/journal.pone.0013139)
Supplement: Table S2 — Average number of proteins (network size) comprised in each network and the number of seeds that are rediscovered during cross-validation when considering different data for generating the specific HIV receptor network. (0.01 MB PDF) [file pone.0013139.s004.pdf]

Table S2: Average number of proteins (network size) comprised in each network and the number of seeds that are re-discovered during cross-validation when considering different data for generating the specific HIV receptor network.

HIV Network Type	Average Network Size	Number of recovered receptors
PPI Network	89 (± 6)	2 of 13
PPI+GO Network	418 (± 8)	11 of 13
PPI+ GO_{enrich} Network	726 (± 16)	12 of 13
