# Supplementary material for: Inference of Surface Membrane Factors of HIV-1 Infection through Functional Interaction Networks
Source: PLoS One. 2010 Oct 12;5(10):e13139. doi: 10.1371/journal.pone.0013139 (PMC2953485; doi:10.1371/journal.pone.0013139)
Supplement: Table S3 — List of functional annotation from Gene Ontology that are used to filter for receptor proteins for the control sets. (0.01 MB PDF) [file pone.0013139.s005.pdf]

Table S3: List of functional annotation from Gene Ontology that are used to filter for receptor proteins for the control sets.

GO Category	GO Term (GO Id)	Definition
**Molecular Function**	Receptor activity (GO:0004872)	Combining with an extracellular or intracellular messenger to initiate a change in cell activity.
	Co-receptor activity (GO:0015026)	Combining with an extracellular or intracellular messenger, and in cooperation with a nearby primary receptor, initiating a change in cell activity
**Biological Process**	Receptor metabolic process (GO:0043112)	The chemical reactions and pathways involving a receptor molecule, a macromolecule that undergoes combination with a hormone, neurotransmitter, drug or intracellular messenger to initiate a change in cell function.
**Cellular Compartment**	Receptor complex (GO:0043235)	Any protein complex that undergoes combination with a hormone, neurotransmitter, drug or intracellular messenger to initiate a change in cell function.
	Membrane (GO:0016020)	Double layer of lipid molecules that encloses all cells, and, in eukaryotes, many organelles; may be a single or double lipid bilayer; also includes. associated proteins.
	Extracellular space (GO:0005615)	That part of a multicellular organism outside the cells proper, usually taken to be outside the plasma membranes, and occupied by fluid.
