# Supplementary material for: Inference of Surface Membrane Factors of HIV-1 Infection through Functional Interaction Networks
Source: PLoS One. 2010 Oct 12;5(10):e13139. doi: 10.1371/journal.pone.0013139 (PMC2953485; doi:10.1371/journal.pone.0013139)
Supplement: Table S4 — Chromosomal location of known and predicted surface membrane proteins. Similar chromosome regions are colored similarly. (0.02 MB PDF) [file pone.0013139.s006.pdf]

Table S4. Chromosomal location of known and predicted surface membrane proteins. Similar chromosome regions are colored similarly.

Known factors	Chrom. Location	Predicted factors	Chrom. Location
CXCR4	2q21	CD2	1q13.1
GPR1	2q33.3	DARC	1q21-q22
ITGA4	2q31.3	HTR6	1p36-p35
CCR9	3p21.3	CSFR3	1p35-p34.3
CCR3	3p21.3	IL1R1	2q12
CCR2	3p21.3	GPR17	2q21
CCR5	3p21.31	CCR1	3p21
CX3CR1	3p21|3p21.3	CCBP2	3p21.3
CXCR6	3p21	RXFP1	4q32.1
CCR8	3p22	GYPB	4q28-q31
APJ	11q12	IL6ST	5q11
CD4	12pter-p12	HTR1B	6q13
DC-SIGN	19p13	HTR1E	6q14-q15
		TNFRSF3	12p13
		GPR182	12q13.3
		RXFP2	13q13.1
		CD79B	17q23
		CD97	19p13
		TNFRSF5	20q12-q13.2
		NPBWR2	20q13.3
		GP1BB	22q11.21-q11.23|22q11.21
