# Supplementary material for: Inference of Surface Membrane Factors of HIV-1 Infection through Functional Interaction Networks
Source: PLoS One. 2010 Oct 12;5(10):e13139. doi: 10.1371/journal.pone.0013139 (PMC2953485; doi:10.1371/journal.pone.0013139)
Supplement: Table S5 — List of seed HIV receptors, including receptor name and type, their functional domains and references indicating an association with HIV-1 infection. (0.07 MB PDF) [file pone.0013139.s007.pdf]

Table S5. List of seed HIV receptors, including receptor name and type, their functional domains and references indicating an association with HIV-1 infection.

Receptor	Receptor type	InterPro domains	References
CD4	Primary receptor for HIV	Ag_CD4, CD4-extracel, Ig-like, Ig-like_fold, Ig_C2-set, Ig_sub, Ig_V-set_sub	[1,2]
CCR5	Co-receptor with CD4	7TM_GPCR_Rhodpsn, CC_5_rcpt	[1-6]
CCR3	Alternative co-receptor with CD4	7TM_GPCR_Rhodpsn, CC_3_rcpt	[4,6,7]
CCR2	Alternative co-receptor with CD4	7TM_GPCR_Rhodpsn, CC_2_rcpt, CC_5_rcpt	[3,4]
CCR8	Alternative co-receptor with CD4	7TM_GPCR_Rhodpsn, CC_8_rcpt	[8,9]
CCR9	Alternative co-receptor with CD4	7TM_GPCR_Rhodpsn, CC_9_rcpt	[10,11]
CXCR4	Alternative co-receptor with CD4	7TM_GPCR_Rhodpsn, CXC_4_rcpt	[1,4]
CXCR6	Co-receptor	7TM_GPCR_Rhodpsn, CXC_6_rcpt	[12,5]
CX3CR1	Co-receptor with CD4	7TM_GPCR_Rhodpsn, CX3C_fract_rcpt	[12-14]
APJ	Alternative co-receptor	7TM_GPCR_Rhodpsn, APJ_rcpt	[11,15-18]
GPR1	Alternative co-receptor	7TM_GPCR_Rhodpsn, GPR1_rcpt	[19-21]
ITGA4	Co-receptor with CD4	Int_alpha_beta-p, Integrin_alpha, Integrin_alpha-2, Integrin_alpha_C	[22-25]
DC-SIGN	Receptor for HIV	AntifreezeII, C-type_lectin.	[26-29]

Receptors are grouped according to their functional domains.

References

1. Alkhatib G, Combadiere C, Broder CC, Feng Y, Kennedy PE, Murphy PM, et al. **CC CKR5: a RANTES, MIP-1alpha, MIP-1beta receptor as a fusion cofactor for macrophage-tropic HIV-1.** *Science*. 1996; **272**(5270):1955–1958.

2. Dragic T, Litwin V, Allaway GP, Martin SR, Huang Y, Nagashima KA, et al. **HIV-1 entry into CD4+ cells is mediated by the chemokine receptor CC-CKR-5.** *Nature*. 1996; **381**(6584):667–673.

3. Magierowska M, Theodorou I, Debré P, Sanson F, Autran B, Rivière Y, et al. **Combined genotypes of CCR5, CCR2, SDF1, and HLA genes can predict the long-term nonprogressor status in human immunodeficiency virus-1-infected individuals.** *Blood*. 1999; **93**(3):936–941.

4. Lee B, Doranz BJ, Rana S, Yi Y, Mellado M, Frade JM, et al. **Influence of the CCR2-V64I polymorphism on human immunodeficiency virus type 1 coreceptor activity and on chemokine receptor function of CCR2b, CCR3, CCR5, and CXCR4.** *J Virol*. 1998; **72**(9):7450–7458.

5. Blaak H, Boers PHM, Gruters RA, Schuitemaker H, Ende MEVD, Osterhaus ADME. **CCR5, GPR15, and CXCR6 are major coreceptors of human immunodeficiency virus type 2 variants isolated from individuals with and without plasma viremia.** *J Virol*. 2005; **79**(3):1686–1700.

6. Agrawal L, Maxwell CR, Peters PJ, Clapham PR, Liu SM, Mackay CR, et al. **Complexity in human immunodeficiency virus type 1 (HIV-1) co-receptor usage: roles of CCR3 and CCR5 in HIV-1 infection of monocyte-derived macrophages and brain microglia.** *J Gen Virol*. 2009; **90**(Pt 3):710–722.

7. Nedellec R, Coetzer M, Shimizu N, Hoshino H, Polonis VR, Morris L, et al. **Virus entry via the alternative coreceptors CCR3 and FPRL1 differs by human immunodeficiency virus type 1 subtype.** *J Virol*. 2009; **83**(17):8353–8363.

8. Utaipat U, Duerr A, Rudolph DL, Yang C, Butera ST, Lupo D, et al. **Coreceptor utilization of HIV type 1 subtype E viral isolates from Thai men with HIV type 1-infected and uninfected wives.** *AIDS Res Hum Retroviruses*. 2002; **18**(1):1–11.

9. Cilliers T, Willey S, Sullivan WM, Patience T, Pugach P, Coetzer M, et al. **Use of alternate coreceptors on primary cells by two HIV-1 isolates.** *Virology*. 2005; **339**(1):136–144.

10. Edwards CTT, Holmes EC, Wilson DJ, Viscidi RP, Abrams EJ, Phillips RE, et al. **Population genetic estimation of the loss of genetic diversity during horizontal transmission of HIV-1.** *BMC Evol Biol*. 2006; **6**:28.

11. Choe H, Farzan M, Konkel M, Martin K, Sun Y, Marcon L, et al. **The orphan seven-transmembrane receptor apj supports the entry of primary T-cell-line-tropic and dualtropic human immunodeficiency virus type 1.** *J Virol*. 1998; **72**(7):6113–6118.

12. Passam AM, Sourvinos G, Krambovitis E, Miyakis S, Stavrianeas N, Zagoreos I, et al. **Polymorphisms of Cx(3)CR1 and CXCR6 receptors in relation to HAART therapy of HIV type 1 patients.** *AIDS Res Hum Retroviruses*. 2007; **23**(8):1026–1032.

13. Garin A, Tarantino N, Faure S, Daoudi M, Lécureuil C, Bourdais A, et al. **Two novel fully functional isoforms of CX3CR1 are potent HIV coreceptors.** *J Immunol*. 2003; **171**(10):5305–5312.

14. Combadiere C, Salzwedel K, Smith ED, Tiffany HL, Berger EA, Murphy PM. **Identification of CX3CR1. A chemotactic receptor for the human CX3C chemokine fractalkine and a fusion coreceptor for HIV-1.** *J Biol Chem*. 1998; **273**(37):23799–23804.

15. Puffer BA, Sharron M, Coughlan CM, Baribaud F, McManus CM, Lee B, et al. **Expression and coreceptor function of APJ for primate immunodeficiency viruses.** *Virology*. 2000; **276**(2):435–444.

16. Zhou N, Zhang X, Fan X, Argyris E, Fang J, Acheampong E, et al. **The N-terminal domain of APJ, a CNS-based coreceptor for HIV-1, is essential for its receptor function and coreceptor activity.** *Virology*. 2003; **317**(1):84–94.

17. Kleinz MJ, Davenport AP. **Emerging roles of apelin in biology and medicine.** *Pharmacol Ther*. 2005; **107**(2):198–211.

18. Zhou N, Fan X, Mukhtar M, Fang J, Patel CA, DuBois GC, et al. **Cell-cell fusion and internalization of the CNS-based, HIV-1 co-receptor, APJ.** *Virology*. 2003; **307**(1):22–36.

19. Shimizu N, Soda Y, Kanbe K, Liu HY, Jinno A, Kitamura T, et al. **An orphan G protein-coupled receptor, GPR1, acts as a coreceptor to allow replication of human immunodeficiency virus types 1 and 2 in brain-derived cells.** *J Virol*. 1999; **73**(6):5231–5239.

20. Tokizawa S, Shimizu N, Hui-Yu L, Deyu F, Haraguchi Y, Oite T, et al. **Infection of mesangial cells with HIV and SIV: identification of GPR1 as a coreceptor.** *Kidney Int*. 2000; **58**(2):607–617.

21. Edinger AL, Hoffman TL, Sharron M, Lee B, O'Dowd B, Doms RW. **Use of GPR1, GPR15, and STRL33 as coreceptors by diverse human immunodeficiency virus type 1 and simian immunodeficiency virus envelope proteins.** *Virology*. 1998; **249**(2):367–378.

22. Wu JQ, Dyer WB, Chrisp J, Belov L, Wang B, Saksena NK. **Longitudinal microarray analysis of cell surface antigens on peripheral blood mononuclear cells from HIV+ individuals on highly active antiretroviral therapy.** *Retrovirology*. 2008; **5**:24.

23. Arthos J, Cicala C, Martinelli E, Macleod K, Ryk DV, Wei D, et al. **HIV-1 envelope protein binds to and signals through integrin alpha4beta7, the gut mucosal homing receptor for peripheral T cells.** *Nat Immunol*. 2008; **9**(3):301–309.

24. Pauls E, Ballana E, Moncunill G, Bofill M, Clotet B, Ramo-Tello C, et al. **Evaluation of the anti-HIV activity of natalizumab, an antibody against integrin alpha4.** *AIDS*. 2009; **23**(2):266–268.

25. Hayes PJ, Miao YM, Gotch FM, Gazzard BG. **Alterations in blood leucocyte adhesion molecule profiles in HIV-1 infection.** *Clin Exp Immunol*. 1999; **117**(2):331–334.

26. Geijtenbeek TBH, Kooyk YV. **DC-SIGN: a novel HIV receptor on DCs that mediates HIV-1 transmission.** *Curr Top Microbiol Immunol*. 2003; **276**:31–54.

27. Masso M. **DC-SIGN points the way to a novel mechanism for HIV-1 transmission.** *MedGenMed*. 2003; **5**(2):2.

28. Kooyk YV, Geijtenbeek TBH. **DC-SIGN: escape mechanism for pathogens.** *Nat Rev Immunol*. 2003; **3**(9):697–709.

29. Geijtenbeek TBH, Kooyk YV. **Pathogens target DC-SIGN to influence their fate DC-SIGN functions as a pathogen receptor with broad specificity.** *APMIS*. 2003; **111**(7-8):698–714.
