# Supplementary material for: Inference of Surface Membrane Factors of HIV-1 Infection through Functional Interaction Networks
Source: PLoS One. 2010 Oct 12;5(10):e13139. doi: 10.1371/journal.pone.0013139 (PMC2953485; doi:10.1371/journal.pone.0013139)
Supplement: Table S6 — List of the potential HIV surface membrane factors inferred by our method, including their functional domains, cell types and references assessing an association with HIV. (0.20 MB PDF) [file pone.0013139.s008.pdf]

Table S6: List of the potential HIV surface membrane factors inferred by our method, including their functional domains, cell types and references assessing an association with HIV.

Receptor	InterPro domains	Cell types	Association with HIV
DARC	Duffy_cmk_rcpt.	High expression: (early) erythroid, endothelial cells	+ [1-3]
CCR1	7TM_GPCR_Rhodpsn, CC_1_rcpt	High expression: whole blood, monocytes, myeloid, dendritic cell	+ [4-6]
CCBP2	7TM_GPCR_Rhodpsn, CXC_4_rcpt	Uniform expression*	+ [7,8]<br>- [9]
CD97	EGF-type_Asp/Asn_hydroxyl_site, EGF_Ca_bd_2, GPCR_2_CD97, GPCR_2_secretin-like, GPS_dom	High expression: CD34, B lymphoblast, dendritic cells, CD8 and CD4 T- cells, NK, myeloid, monocytes	+ [10]
GP1BB	LRR-contain_N, Cys-rich_flank_reg_C	High expression: CD34, monocytes and whole blood	?
HTR6	7TM_GPCR_Rhodpsn	Uniform expression	+
HTR1B	5HT1B_rcpt, 7TM_GPCR_Rhodpsn	Uniform expression	?
HTR1E	5HT1F_rcpt, 7TM_GPCR_Rhodpsn	Uniform expression	?
RXFP2	7TM_GPCR_Rhodpsn, LDL_rcpt_classA_cys-rich_rpt, Leu-rich_rpt, LRR-contain_N, Leu-rich_rpt_typical-subtyp, Relaxin_rcpt	Low expression	?
RXFP1	7TM_GPCR_Rhodpsn, LDL_rcpt_classA_cys-rich, Leu-rich_rpt, LRR-contain_N, Leu-rich_rpt_typical-subtyp, Relaxin_rcpt	No expression profiles available	?
GPR17	7TM_GPCR_Rhodpsn, P2_purnocptor	Uniform expression	?
GPR182	7TM_GPCR_Rhodpsn, G10D_rcpt	Uniform expression	?
NPBWR2	7TM_GPCR_Rhodpsn, Neuropept_W_rcpt	Uniform expression	- [11]
GYPB	Glycophorin	High expression: (early) erythroid and endothelial cells	?
CD2	Ag_CD2, Ig-like_fold, Ig_C2-set, Ig_V-set, T-cell_sdhesion_molc_CD2.	High expression: dendritic, myeloid, monocytes, NK, CD8 and CD4 T cells, whole blood	+ [12-15]

CSF3R	FN_III, Hematopoietin_rcpt_gp130_CS, IgC2-like_lig-bd	High expression: myeloid cells, monocytes and whole blood	+ [16,17]
IL1R1	Ig, Ig-like, Ig-like_fold, Ig_sub, IL1_rcpt_1, IL1R_rcpt	No expression profile available	- [18,19]
CD79B	Ig-like, Ig-like_fold, Ig_sub, Ig_V-set, Phos_immunorcpt_sig_ITAM.	High expression: CD34, endothelial and dendritic cells	+ [13,20]
IL6ST	FN_III, Hematopoietin_rcpt_gp130_CS, Ig-like_fold, IgC2-like_lig-bd	Uniform expression	+ [21-23]
TNFRSF5	Fas_rcpt, TNFR_Cys_rich_reg	High expression: B lymphoblasts	+ [24-26]
TNFRSF3	TNFR_3_LTBR, TNFR_Cys_rich_reg	High expression: myeloid, monocytes and whole blood	+ [27]

(*): CD34, endothelial, B lymphoblasts, dendritic, myeloid, monocytes, NK, CD8 and CD4 T cells, whole blood

(+) Predictions with literature supporting an interaction with HIV are marked as (+).

(-) Indicates predictions with negative evidences.

(?) For predictions without literature on interaction or non-interaction the association remains unclear.

References

1. Lachgar A, Jaureguiberry G, Buenac HL, Bizzini B, Zagury JF, Rappaport J, et al. **Binding of HIV-1 to RBCs involves the Duffy antigen receptors for chemokines (DARC).** *Biomed Pharmacother*. 1998; **52**(10):436–439.

2. Walton RT, Rowland-Jones SL. **HIV and chemokine binding to red blood cells–DARC matters.** *Cell Host Microbe*. 2008; **4**(1):3–5.

3. He W, Neil S, Kulkarni H, Wright E, Agan BK, Marconi VC, et al. **Duffy antigen receptor for chemokines mediates trans-infection of HIV-1 from red blood cells to target cells and affects HIV-AIDS susceptibility.** *Cell Host Microbe*. 2008; **4**(1):52–62.

4. Shimizu N, Tanaka A, Oue A, Mori T, Ohtsuki T, Apichartpiyakul C, et al. **Broad usage spectrum of G protein-coupled receptors as coreceptors by primary isolates of HIV.** *AIDS*. 2009; **27**(7):761–769.

5. McKnight A, Dittmar MT, Moniz-Periera J, Ariyoshi K, Reeves JD, Hibbitts S, et al. **A broad range of chemokine receptors are used by primary isolates of human immunodeficiency virus type 2 as coreceptors with CD4.** *J Virol*. 1998; **72**(5):4065–4071.

6. Utaipat U, Duerr A, Rudolph DL, Yang C, Butera ST, Lupo D, et al. **Coreceptor utilization of HIV type 1 subtype E viral isolates from Thai men with HIV type 1-infected and uninfected wives.** *AIDS Res Hum Retroviruses*. 2002; **18**(1):1–11.

7. Neil SJD, Aasa-Chapman MMI, Clapham PR, Nibbs RJ, McKnight A, Weiss RA. **The promiscuous CC chemokine receptor D6 is a functional coreceptor for primary isolates of human immunodeficiency virus type 1 (HIV-1) and HIV-2 on astrocytes.** *J Virol*. 2005; **79**(15):9618–9624.

8. Shimizu N, Tanaka A, Oue A, Mori T, Apichartpiyakul C, Hoshino H. **A short amino acid sequence containing tyrosine in the N-terminal region of G protein-coupled receptors is critical for their potential use as co-receptors for human and simian immunodeficiency viruses.** *J Gen Virol*. 2008; **89**(Pt 12):3126–3136.

9. Nibbs RJ, Wylie SM, Yang J, Landau NR, Graham GJ. **Cloning and characterization of a novel promiscuous human beta-chemokine receptor D6.** *J Biol Chem*. 1997; **272**(51):32078–32083.

10. Zhou H, Xu M, Huang Q, Gates AT, Zhang XD, Castle JC, et al. **Genome-scale RNAi screen for host factors required for HIV replication.** *Cell Host Microbe*. 2008; **4**(5):495–504.

11. Efremov R, Truong MJ, Darcissac EC, Zeng J, Grau O, Vergoten G, et al. **Human chemokine receptors CCR5, CCR3 and CCR2B share common polarity motif in the first extracellular loop with other human G-protein coupled receptors implications for HIV-1 coreceptor function.** *Eur J Biochem*. 1999; **263**(3):746–756.

12. Meuer SC, Hussey RE, Fabbi M, Fox D, Acuto O, Fitzgerald KA, et al. **An alternative pathway of T-cell activation: a functional role for the 50 kd T11 sheep erythrocyte receptor protein.** *Cell*. 1984; **36**(4):897–906.

13. Shen A, Yang H, Zhou Y, Chase AJ, Boyer JD, Zhang H, et al. **Novel pathway for induction of latent virus from resting CD4⊕T cells in the simian immunodeficiency virus/macaque model of human immunodeficiency virus type 1 latency.** *J Virol*. 2007; **81**(4):1660–1670.

14. Bressler P, Pantaleo G, Demaria A, Fauci AS. **Anti-CD2 receptor antibodies activate the HIV long terminal repeat in T lymphocytes.** *J Immunol*. 1991; **147**(7):2290–2294.

15. Wu JQ, Dyer WB, Chrisp J, Belov L, Wang B, Saksena NK. **Longitudinal microarray analysis of cell surface antigens on peripheral blood mononuclear cells from HIV+ individuals on highly active antiretroviral therapy.** *Retrovirology*. 2008; **5**:24.

16. Dunn SJ, Khan IH, Chan UA, Scearce RL, Melara CL, Paul AM, et al. **Identification of cell surface targets for HIV-1 therapeutics using genetic screens.** *Virology*. 2004; **321**(2):260–273.

17. Rapaport R, McLean C, Campbell T. **Filgrastim Stimulates HIV-1 Replication in Monocytoid Cells..** In: *Program Abstr Conf Retrovir Oppor Infect 11th 2004 San Franc Calif.*; 2004.

18. Wang C, Song W, Lobashevsky E, Wilson CM, Douglas SD, Mytilineos J, et al. **Cytokine and chemokine gene polymorphisms among ethnically diverse North Americans with HIV-1 infection.** *J Acquir Immune Defic Syndr*. 2004; **35**(5):446–454.

19. Do H, Vasilescu A, Carpentier W, Meyer L, Diop G, Hirtzig T, et al. **Exhaustive genotyping of the interleukin-1 family genes and associations with AIDS progression in a French cohort.** *J Infect Dis*. 2006; **194**(11):1492–1504.

20. Nance CL, Shearer WT. **SDF-1alpha regulates HIV-1-gp120-induced changes in CD79b surface expression and Ig production in activated human B cells.** *Clin Immunol*. 2002; **105**(2):208–214.

21. Macal M, Sankaran S, Chun T, Reay E, Flamm J, Prindiville TJ, et al. **Effective CD4+ T-cell restoration in gut-associated lymphoid tissue of HIV-infected patients is associated with enhanced Th17 cells and polyfunctional HIV-specific T-cell responses.** *Mucosal Immunol*. 2008; **1**(6):475–488.

22. Brenchley JM, Paiardini M, Knox KS, Asher AI, Cervasi B, Asher TE, et al. **Differential Th17 CD4 T-cell depletion in pathogenic and nonpathogenic lentiviral infections.** *Blood*. 2008; **112**(7):2826–2835.

23. Ndhlovu LC, Chapman JM, Jha AR, Snyder-Cappione JE, Pagán M, Leal FE, et al. **Suppression of HIV-1 plasma viral load below detection preserves IL-17 producing T cells in HIV-1 infection.** *AIDS*. 2008; **22**(8):990–992.

24. Vanham G, Penne L, Devalck J, Kestens L, Colebunders R, Bosmans E, et al. **Decreased CD40 ligand induction in CD4 T cells and dysregulated IL-12 production during HIV infection.** *Clin Exp Immunol*. 1999; **117**(2):335–342.

25. Martin G, Roy J, Barat C, Ouellet M, Gilbert C, Tremblay MJ. **Human immunodeficiency virus type 1-associated CD40 ligand transactivates B lymphocytes and promotes infection of CD4+ T cells.** *J Virol*. 2007; **81**(11):5872–5881.

26. Maurais E, Cantin R, Tremblay MJ. **Human immunodeficiency virus type 1-anchored CD40 ligand induces secretion of the chemokine interleukin-8 by human primary macrophages.** *Virology*. 2009; **385**(1):227–232.

27. Marshall WL, Brinkman BM, Ambrose CM, Pesavento PA, Uglialoro AM, Teng E, et al. **Signaling through the lymphotoxin-beta receptor stimulates HIV-1 replication alone and in cooperation with soluble or membrane-bound TNF-alpha.** *J Immunol*. 1999; **162**(10):6016–6023.
