# Supplementary material for: Inference of Surface Membrane Factors of HIV-1 Infection through Functional Interaction Networks
Source: PLoS One. 2010 Oct 12;5(10):e13139. doi: 10.1371/journal.pone.0013139 (PMC2953485; doi:10.1371/journal.pone.0013139)
Supplement: Text S1 — Definition of functional similarity and description of the different HIV receptor network types generated from different functional data, including a detailed performance comparison of their ability to infer novel surface membrane factors interacting with HIV-1. (0.06 MB PDF) [file pone.0013139.s009.pdf]

Supporting Information

Functional similarity of proteins

We determine the functional similarity between two proteins by analyzing their GO annotations using semantic similarity. We first compute the similarity of two GO terms and extend the measure to determine the functional similarity of two proteins annotated with several GO terms. Note, the functional similarity between two proteins is computed separately for each of the GO subontologies: molecular function (MF), biological process (BP) and cellular component (CC).

Semantic similarity between GO terms

To compute the semantic similarity between two GO terms we use the approach proposed by Lin [1]. Following Lin's definition, the information content of a GO term t is defined as follows:

$$IC(t) = -log\left(\frac{freq(t)}{freq(root)}\right), \tag{1}$$

where the frequency of a term is defined as the number of times a term or any of its descendants occurs. Thus, less frequent terms and terms with few occurring descendants are considered more informative.

Based on this measure, the semantic similarity between two terms is defined as the ratio of the information content of their most informative common ancestor and the information contents of both concepts [1]. The information content of the most informative common ancestor is given by:

$$shareIC(t_1, t_2) = max\left\{IC(t) | t \in CA(t_1, t_2)\right\}, \tag{2}$$

where $CA(t_1, t_2)$ is the set of all common ancestors between terms t_1 and t_2. The similarity between two terms is then defined as:

$$sim(t_1, t_2) = \frac{2 * shareIC(t_1, t_2)}{IC(t_1) + IC(t_2)}. \tag{3}$$

Semantic similarity between proteins

The semantic similarity between proteins is determined based on the similarity of their associated GO terms. Since often proteins are annotated with more than one term, the similarity of a protein p to a group g of terms is defined as the average similarity of its terms to their most similar terms in g [2] (where $t(p)$ is the set of terms annotated to protein p):

$$Sim(p, g) = \frac{\sum\limits_{t_1 \in t(p)} max\left\{sim(t_1, t_2) | t_2 \in g\right\}}{|t(p)|} \tag{4}$$

Finally, the functional GO similarity between two proteins is defined as the average similarity of their GO terms:

$$GO_{Sim}(p_1, p_2) = \frac{Sim(p_1, t(p_2)) + Sim(p_2, t(p_1))}{2}. \tag{5}$$

GO_{Sim} ranges between 0 and 1 depending on the similarity of the GO annotations between two proteins, whereby 1 indicated functional equality and 0 indicates maximal functional distance. The functional similarity of all three GO subontologies is added and then averaged to obtain an overall similarity score for two proteins:

$$GO_{Sim}(p_1, p_2) = \frac{GO_{Sim_{\mathrm{MF}}}(p_1, p_2) + GO_{Sim_{\mathrm{BP}}}(p_1, p_2) + GO_{Sim_{\mathrm{CC}}}(p_1, p_2)}{3}. \tag{6}$$

Impact of the functional data on the outcomes of the prediction methods

We use protein interaction data and functional annotations to generate an HIV specific receptor network. In addition, we assessed the influence of using manually curated and predicted functional annotation on our prediction method by applying it to differently compiled HIV networks.

HIV network types

First, we only considered proteins that interact directly with any seed receptors when generating the specific HIV receptor network which will be called PPI network. Next, we integrated proteins that interact directly with any seed and all proteins which are functionally very similar to any seed considering only manually curated functions – PPI–GO network. Third, we consider interaction data in combination with enriched functional annotation (manual curated and predicted function) – PPI–GO_{enrich} network.

Performance comparison of the HIV network types

We compare the ability of our frameworf to find novel surface membrane factors within the three different HIV networks by using cross-validation. Leave-one-out cross-validations are performed over the 13 known HIV receptors for the PPI, PPI–GO and PPI–GO_{enrich} networks. For cross-validation, we remove one known HIV receptor from the initial list and try to re-discover this receptor by means of our method. We build an HIV receptor network by considering only the remaining receptors as seeds and rank the proteins according to their centrality within the network. Subsequently, we determine whether the left-out receptor is re-discovered and at which position of the ranked list. We repeat this procedure for each seed and determine an average recovery rate across all receptors and for each network type. Table S1 shows the average network size and the number of recovered (hidden) seeds for the three different kinds of HIV-receptor networks.

The seed re-discovery rate is very low when using only protein interaction data. Only two out of 13 receptors can be captured within the generated networks. This rate increases significantly up to 11 and 12 detected receptors when considering additionally functional annotation (PPI–GO) as well as predicted functions (PPI–GO_{enrich}), respectively. Two receptors are not covered in the PPI–GO networks, namely DC-SIGN and ITGA4, whereas the latter one is also not detected using the enriched network, most likely due to different ligands and a lower functional similarity to the other seed receptors. Figure S1 shows the seed re-discovery rate using the three network types and their distribution across the ranked lists. In general, the number of re-discovered receptors is relatively low when considering only the top ranked proteins (e.g. x = 5%). However, the recovery rate increases significantly the more proteins of the respective networks are examined (except for PPI), until it converges to the total number of detected seeds displayed in Table S1. Figure S1 emphasizes the improved performance of our method when using interaction and functional data and underlines the value of functional data for capturing relevant receptors and surface membrane factors in the HIV network. Protein interaction data alone is not sufficient for finding the known receptors, since it captures similar ligands rather then functionally similar receptors. Utilizing interaction and functional annotations allows to generate more complete networks in biological sense. This is reflected in the average network size of the different network types which increases from 89 proteins to 418 and 726 for PPI–$GOGO_{enrich}$.

Comparing the recovery rates of PPI–GO and PPI–GOenrich across the ranked list clearly shows that 'hidden' receptors are better recovered and more highly ranked within the enriched than in the non-enrich network. However, the superior performance might result from the larger size of the enriched networks, e.g. the number of proteins that is considered at the different x is twice as high for PPI–GO_{enrich} because the networks are in average about two times larger. To ensure that the higher recovery rate is not affected by the larger amount of proteins we normalize the recovery rates by the number of proteins considered at each rank x. Original and normalized recovery rates for PPI–GO and PPI–GO_{enrich} are compared in Figure S2. There is a higher fraction of known receptors among the top ranked proteins for

PPI–GO$_{enrich}$ especially for top ranks from $x = 1\%$ to 3%. This continues till $x = 20\%$ and reverses afterwards. Normalized and original cross-validation outcomes underline the significance of utilizing not only interaction and (manual) functional data but also predicted functional annotations for our purpose.

References

1. Lin D (1998) An information-theoretic definition of similarity. In: Proceedings of the 15th ICML. Madison WI, pp. 296–304.

2. Couto FM, Silva MJ, Pedro Coutinho PM (2007) Measuring semantic similarity between gene ontology terms. Data Knowl Eng 61: 137–152.
